# The psychosocial experiences of pregnant women in the early stages of the COVID-19 pandemic: A retrospective qualitative study

**DOI:** 10.1371/journal.pone.0299219

**Published:** 2024-02-28

**Authors:** Abigail Kusi Amponsah, Edward Appiah Boateng, Jerry Armah, Joana Kyei Dompim, Douglas Gyamfi, Alberta Lomotey, Faithful Adwoa Annobil, Amena Ekua Amankrah, Rifka Abdallah Youshah, Elizabeth Uzoka Beauty, Francis Diji, Victoria Bam

**Affiliations:** 1 Department of Nursing, Kwame Nkrumah University of Science and Technology, Kumasi, Ghana; 2 Department of Nursing Sciences, University of Turku, Turku, Finland; 3 Department of Obstetrics and Gynaecology, Komfo Anokye Teaching Hospital, Kumasi, Ghana; 4 St. Martins Catholic Hospital, Kumasi, Ghana; ICMR - National Institute for Research in Reproductive and Child Health (ICMR-NIRRCH), INDIA

## Abstract

**Background:**

Pregnant women are among the most vulnerable and suffer the most during pandemics, according to earlier studies. Pregnant women had to seek healthcare for both themselves and their unborn child(ren) in the wake of the COVID-19 pandemic, which was unprecedented. Pregnant women’s psychosocial experiences during pandemics are crucial since they both directly and indirectly affect the course of pregnancy and childbirth. The study therefore sought to explore the psychosocial experiences of pregnant women during the COVID-19 pandemic.

**Methods:**

In this retrospective qualitative study, 15 nursing mothers who were attending a postnatal clinic at the Kwame Nkrumah University of Science and Technology (KNUST) hospital in Ghana were recruited. Individual interviews were conducted with mothers who were pregnant between March and December 2020. The audio-recorded interviews were transcribed verbatim and inductively analysed into themes.

**Results:**

Nursing mothers were aged 25–30 years and had infants ranging from 5 months to 15 months. Thirteen (13) were married and two were single. Two (2) major themes and five (5) subthemes emerged from the study. The unpleasant feelings connected to the potential for contracting COVID-19 and experiencing stress were described by the theme, “Fear and Stress”. Participants’ social experiences (support from significant others), alterations in daily routine and the economic impact because of the pandemic were presented as the “Socioeconomic impact”.

**Conclusion:**

Pregnant women go through several challenges during pregnancy such as perceived stress and anxiety. These are likely to heighten during a pandemic, as presented in the study. They therefore need emotional and psychosocial support in such uncertain times to improve outcomes of pregnancy.

## 1. Introduction

The Coronavirus Disease of 2019 (COVID-19), caused by severe acute respiratory syndrome coronavirus 2 (SARS-CoV-2) created a new approach to everyday life which we are not really used to, like the lockdown restrictions, wearing of nose masks and other safety protocols [1]. These safety protocols prescribed by the World Health Organization (WHO) and the Ministries of Health locally were not necessarily comfortable for everyone including expectant mothers [2].

Pregnancy has been compared by some women to an emotional roller coaster [3]. Even for women who are having a normal pregnancy, the dread of the unknown is a big issue for the majority of pregnant women [4]. One can imagine how pregnant women must have felt during the COVID-19 pandemic. The COVID-19 pandemic is an extremely stressful event that has caused numerous disruptions, including loss of housing and money, physical and social isolation, and fear of the infection [5, 6]. In addition to all of these, are some problems making pregnant women more anxious. These include a higher risk of getting COVID-19, being vulnerable to severe consequences, having a higher chance of dying, having a larger chance of mother-to-child transmission, and COVID-19’s potential to affect the foetus [7].

Studies show that in previous epidemics, pregnant women have been among the most vulnerable groups of people and have suffered badly [8–10]. Pregnant women in Hong Kong during the COVID-19 pandemic reported feeling frustrated, anxious, having trouble sleeping, and having their daily routines disrupted [11]. Likewise studies conducted in South Africa [12, 13], Zambia [14] and Ethiopia [15] reported higher rates of anxiety, depression and overall psychological problems found among pregnant women. Women were reluctant to visit health facilities during the Ebola epidemic out of fear of getting sick [16]. Furthermore, while medical facilities are required to treat all patients, there were increased precautions and protocols in place to care for pregnant patients amid concerns of potential virus transmission [16]. It was expected that pregnant women would face similar challenges during the COVID-19 pandemic as the general population. Worry, anxiety, and fear during pregnancy are unhealthy for both the expectant mother and the developing foetus [11].

Pregnant women are considered a uniquely vulnerable group because of their compromised immunological functions, cardiopulmonary changes, altered physiology and susceptibility to infections [17]. In a global survey of 2,100 pregnant women, those who contracted COVID-19 while pregnant had a 20-fold higher risk of dying than those who did not [18]. Studies have reported that, vertical transmission from mother to foetuses within the uterus does happen, although it is uncommon [19]. Additionally, most newborns from mothers infected with COVID-19 do not appear to contract the infection after birth. However, there have been reported cases of new-borns with viraemia and subsequent neurological issues [20, 21]. During infectious disease epidemics like the current COVID-19 pandemic, pregnant women may experience higher levels of stress, anxiety, and depression. However, research on the psychosocial experiences of pregnant women during the COVID-19 pandemic remains limited in Ghana and insufficiently explored.

The current study therefore sought to assess the psychosocial experiences of pregnant women in the early stages of the COVID-19 pandemic.

## 2. Materials and methods

### 2.1 Study design

The study adopted a retrospective qualitative approach because it was deemed appropriate in exploring the psychosocial experiences of postnatal mothers since they had lived that experience [22].

### 2.2 Study setting and population

The study was conducted among women at the postnatal clinic of the KNUST Hospital in Ghana. The university hospital started as a dressing station in 1952. It has since expanded through alterations and extensions to become a fully operational hospital with 125 beds. Currently, the hospital serves students, employees and dependents, and residents from around 30 nearby communities. Every week, more than 80 women visit the hospital’s postnatal clinic.

### 2.3 Sampling and sample size

A purposive sampling was adopted in selecting participants who met the inclusion criteria for the study. The inclusion criteria were (1) Women who were 18 years and above, with an infant of age from 2 to 17 months. The assumption was that a woman who was pregnant during October 2020 spent the first trimester during the period of interest and would have had a child aged 2 months as at the time of data collection, that is August 2021. Also, a woman who delivered in March 2020 would have a child aged seventeen (17) months. (2) Women who attended the postnatal clinic of KNUST hospital, (3) Women who could speak English or Asante Twi (a local Ghanaian language). Sample size was determined by data saturation, where no new data were generated from the interviews conducted.

### 2.4 Data collection and analysis

A semi-structured interview guide was employed in the data collection process. The interview guide was designed based on the study’s objectives, reviewed, and validated by two experts in qualitative research of maternal health. The guide was pilot tested with 5 women who met the inclusion criteria at the postnatal clinic of Kumasi South Hospital. The outcome of the pilot influenced modifications to the guide before utilized for data collection. The entire study commenced on July 2021 and ended in December 2021. Participants recruited were engaged in a face-to-face interview by one researcher, and an assistant who recorded the interviews and took notes. Interviews were conducted in the conference room of the hospital and lasted for about 20 minutes per session. The interviewer was a mixed-methods researcher with 8 years of experience in qualitative research of maternal and paediatric health. Caution was taken by the interviewer to ensure there was minimal probing into participants’ private lives. This caution was exercised by having a well-designed interview guide focused on the research objectives and the interviewer demonstrated proficiency in effectively manoeuvring through the interviews. This was to control instances where participants may disclose private matters which may be sensitive and unrelated to the research objectives. All COVID-19 safety protocols such as social distancing and wearing nose masks were adhered to.

The audiotaped interviews were transcribed verbatim in Microsoft Word. Transcribed data was read over continuously to ensure that exact statements of participants were captured in text. Braun and Clarke [23] reflexive thematic analysis guided the process of analysis, which began with an open coding phase where 4 researchers (AKA, JA, FAA and AEA) independently read through the data to identify initial codes without pre-set categories. Subsequently, axial coding followed where the team of 4 (AKA, JA,FAA and AEA) systematically linked initial codes, collaboratively identifying patterns and ensuring coding reliability and validity through cross-verification. This phase led to the creation of a code book, cataloguing all codes with descriptions, which served as a continual reference. The researchers then grouped coherent codes into subthemes, interpreting underlying meanings and connections, with each subtheme reflecting distinct yet related data segments. These subthemes were further abstracted into broader themes, capturing overarching patterns and narratives in the data, comprising key participant experiences and perspectives. Identified themes and subthemes were revised to confirm that they were supported by the corresponding participant quotes.

### 2.5 Ethical consideration

Ethical approval was obtained from the Committee on Human Research Publication and Ethics (CHRPE) of the KNUST School of Medical Sciences with reference number “CHRPE/AP/403/21”. Administrative approval from the university hospital was also obtained as well as the respective in-charge of the postnatal clinic. All participants took part in the study after the details of the study was thoroughly explained to them. A written informed consent was obtained from each participant. They were made aware of the possibility to opt out of the study at any point in time if they wished. Participants quotes were presented as that of anonymous individuals to ensure confidentiality.

### 2.6 Rigour

Lincoln and Guba [24] evaluative criteria were adopted in ensuring rigour in the study. Credibility was achieved by ensuring that the interviewer had the required experience, knowledge, and skills to conduct that role. Also, there was evidence of engagement of participants through the provision of participants’ quotes. Transferability was achieved by providing sufficient data on participant characteristics, the study setting, and processes involved in collecting and analyzing the study’s findings. Dependability was achieved by ensuring consistency in the data collection (one interviewer using the same interview guide) and analysis procedures, as well as maintaining a transparent documentation throughout the research process. Confirmability in our study was achieved through the method of triangulation. This process involved engaging multiple researchers who independently analysed the study data and had discussion with each other to enhance the data interpretations.

## 3. Results

### 3.1 Sociodemographic data

Participants were aged from 25–30 years, and had infants aged from 3 months to 15months. Thirteen (13) of them were married and majority (n = 11) were employed. Most of the participants were multiparous (n = 10) and all of the participants recruited (n = 15) were Christians. The data is displayed in **Table 1** below;

**Table 1:** Sociodemographic characteristics of participants.

Participant ID	Age (years)	Age of infant (months)	Marital status	Employment status	Religion	Parity
R1	28	4	Single	Unemployed	Christian	3
R2	26	5	Married	Employed	Christian	1
R3	28	7	Married	Employed	Christian	3
R4	26	2	Married	Employed	Christian	1
R5	25	11	Married	Employed	Christian	2
R6	28	3	Married	Unemployed	Christian	1
R7	25	10	Single	Employed	Christian	2
R8	29	6	Married	Employed	Christian	1
R9	30	10	Married	Employed	Christian	3
R10	30	12	Married	Employed	Christian	2
R11	29	9	Married	Employed	Christian	2
R12	30	6	Married	Employed	Christian	1
R13	27	8	Married	Unemployed	Christian	2
R14	29	15	Married	Employed	Christian	3
R15	29	11	Married	Unemployed	Christian	2

### 3.2 Themes

Two (2) major themes and five (5) subthemes emerged from the study (refer to **Table 2**).

**Table 2:** Themes and subthemes.

THEMES	SUBTHEMES
Fear and Stress	• Fear of contracting COVID-19 • Stress during the period
Socioeconomic impact	• Support from significant others • Effect on daily routine • Economic impact

### 3.3 Fear and stress

This theme presented the unpleasant emotional states and stress of participants that evolved because of the pandemic. Two (2) subthemes evolved from this theme: Fear of contracting COVID-19 and Stress during the period.

#### 3.3.1 Fear of contracting COVID-19

Participants perceived the virus as high risk to their health. The fear was generated mainly from the numerous deaths recorded from foreign countries. Also, they hoped the virus never reached Ghana, so news about the country recording several hundreds to thousands of cases and deaths heightened fear levels. They viewed the disease as serious and anticipated getting infected if they left their residence or downplayed the social distancing protocols. This was evident in their quotes as they reported;

*“We all felt bad because of the death that was happening at the outside and even within Ghana here it wasn’t easy*.*”* **(R5)**

*“It was quite scary since I knew nothing about it and lots of people were dying*. … *We were all praying it won’t reach that point but before we knew all of a sudden it was like getting to thousand there about*. *We were all hoping that it won’t reach Ghana…It was scary when I heard people were dying because of the virus”* **(R8)**

*“I was really scared because the media was reporting that most Italians were dying of the pandemic*. *The thought of it always gave me headaches seriously*.*”* **(R13)**

#### 3.3.2 Stress during the period

Participants revealed that the COVID-19 pandemic was one of the most stressful life events they have ever faced. According to participants, COVID-19 introduced a new system of life that involved so many restrictions to protect oneself, especially with the wearing of nose masks and washing hands frequently. They believed pregnancy itself was a stressful life event both physically, physiologically and emotionally, so the pandemic compounded the stress they faced. Emotional stress for instance was predominant among participants’ whose partners were not around amidst the pandemic.

*“I would say it was very stressful because of the restrictions or safety protocols*. *Not going to places*, *washing hands frequently and especially wearing the nose mask*.*”* **(R12)**

*“This pregnancy was really stressful because one had to be always covering one’s nose and mouth*, *washing hands almost every time and distancing oneself from people all the time*. *But generally speaking*, *I’ll say all these protocols also made me very hygienic and protected me from other sicknesses*, *not only COVID-19*.*”* **(R13)**

*“I’d say pregnancy amidst COVID-19 was very stressful because*, *my husband could not come down to Ghana which put emotional stress on me*. *Wearing of the nose masks too was a big deal for me”* **(R14)**

### 3.4 Socioeconomic impact

This theme described the impact the pandemic had on both their social and economic lives. Three (3) subthemes evolved from this theme; Support from significant others, Effect on daily routine and Economic impact.

#### 3.4.1 Support from significant others

It was evident that interpersonal relations and routines were affected. The impact was however distinct for most individuals, because as some participants had family around often to support them, others had little to no support due to the travel restrictions. This theme assessed mainly their support system during the pandemic. Participants said they had very robust support mostly with the activities of daily living, from family and friends during the period. This translated into having many familiar faces around hence, they were not lonely. The support ranged from just having the husband or nuclear family around to the bigger households with the external family around. This was evident in some quotes;

*“They were very supportive because going out and coming in*, *washing all your hands*, *with the nose mask*, *sanitizer they want to make sure everything is intact*. *Before I step out and when coming back*, *they want me to be okay before like when I enter in the house*, *they want me to wash my hands*, *sanitize my hands before I touch anything”*. **(R4)**

*“My family was around*. *They buy food stuff in the house*, *so at least if you don’t have much money on you*, *you can just take some*. *We were just cooking*. *I got support from friends especially at church*. *At church I quite remember they provided rice and oil*. *They also did well*. **(R8)**

*“In fact*, *I had support from no family member because none of them wanted to travel to Kumasi’s sake of fear of contracting the disease*. *My husband too couldn’t come because airports were closed*. *I was very lonely and even bled at a point but no one was there to help*. *Because the midwives had given us their contacts*, *I had to call one of them to teach me what to do before reporting to the hospital*.*”* **(R14)**

#### 3.4.2 Effect on daily routine

On the basis of their daily routines, most participants reported that it was unaffected. This was mainly because most of them had already resorted to staying at one place as a result of the pregnancy. Therefore, introduction of various restrictions did little to rattle them.

*“I’m an indoor person so it like it didn’t change anything just going to the hospital coming back*, *going to work that’s all*, *yeah”*. **(R4)**

*“The doctor ordered me not to do anything in the house because of my pregnancy complications so I was always in bed*. *Even when I wanted to bath*, *they had to carry my water into the bathroom*. *In view of this*, *I will not say the pandemic affected my normal daily activities”*. **(R11)**

*“Because I lost my job as a result of the pandemic*, *I was always home doing normal house hold chores like cleaning*, *cooking*, *washing and taking care of my first child*. *I’ll say the pandemic positively affected my normal daily activities”*. **(R13)**

#### 3.4.3 Economic impact

The pandemic capsized the economy of most countries. Ghana was no exception and the citizens were the most affected. The restrictions prevented most people from going to work and many other businesses closed down. Other companies had to lay off workers to keep up with the economic fallout. Participants reported that they were heavily affected by the pandemic and the consequent introduction of restrictions especially during the lockdown period. The responses include;

*“Because our work got spoilt*, *I had to come and stay home and till now*, *I’ve not had another job*. *I’ll say the pandemic has rendered me unemployed”*. **(R13)**

*“Sake of the pandemic I had to close down my shop for the fear of contracting it from a client*. *Before I even closed down the shop*, *client number had reduced drastically because people were not going out*, *hence no need for make-overs”*. **(R14).**

Participants also verbalized that their financial support came from their partners and family members even before the onset of COVID-19, and the government payroll some were on contributed to them being less affected.

“*I was solely depending on my husband*. *He was the one doing everything in terms of finances*”. **(R11)**

*“Oh*! *Like I said*, *I’m a government school teacher so the government was still paying us at the end of every month even as we were home*. *My husband too was fulfilling his financial commitment to me*. **(R12)**

## 4. Discussion

The current study sought to explore psychosocial experiences of pregnant women during the early stages of the COVID-19 pandemic. Fear and stress among the general population during the COVID-19 pandemic have been extensively studied and documented in the existing literature [25, 26]. According to these studies, the pandemic had a profound impact on individuals, including pregnant women’s mental well-being, leading to increased levels of fear across various populations [25–28]. These reports are consistent with our findings which revealed reports of fear among pregnant women. As the pandemic significantly impacted most nations, pregnant women experienced heightened levels of fear due to the inherent challenges and uncertainties of pregnancy. In the absence of a pandemic, several factors contribute to fear during pregnancy. Some of these factors are health concerns such as worries about birth defects, complications, unpleasant experience with previous pregnancy, lack of social support and financial stress [29–33]. On the other hand, the pandemic also brought with it a barrage of fears, uncertainties, and undue hardships amongst pregnant women [34]. Participants viewed COVID-19 as serious and anticipated getting infected if they left their residence or downplayed the social distancing protocols. They feared its impact on their health. Although there are reports of fear during pregnancy in the presence or absence of a pandemic, the difference lies in the experiences. Fear is compounded in pregnancy during a pandemic considering that pregnant women must deal with the uncertainties of pregnancy and fear of infection from the pandemic as reported by our study. Also, our finding was consistent with studies of Fallon, et al. [35], Oluoch-Aridi, et al. [36] and Dahl, et al. [37]where participants perceived a high risk and possible mortality, and therefore undertook the necessary safety precautions such as using sanitizer and washing their hands regularly and wearing face masks. These participants were negatively impacted by the rise in fear because they were more prone to entertain negative thoughts than feel at peace. Even though most of the participants were multiparous, there were reports of fear. It could be suggested that their previous pregnancy experiences may not have had much impact in managing fear and their concerns, also suggested by Ravaldi, et al. [38]. This is because it was most likely their first time being pregnant during a pandemic and missed out on the normal pregnancy experience [39]. In addition to that, public health education at healthcare facilities on how pregnant women were at a higher risk of COVID-19 infection [40, 41] may have contributed to the rising fear. On the positive side, pregnant women were more serious about taking preventative steps to stop the disease from spreading because of their concern of developing COVID-19. This concern and the steps to taking preventive measures may have also been as a result of the health education they received during their prenatal visits [41].

On the issue of stress, participants reported stressful situations predisposing them to higher levels of anxiety and ineffective coping. The stress mainly stemmed from the introduction of restrictions and economic hardships. Our findings were consistent with studies by Puertas-Gonzalez, et al. [42] and Mortazavi and Ghardashi [11]. Puertas-Gonzalez, et al. [42] compared stress levels between pregnant women in the COVID-19 period and those before the pandemic. In their study, the group who were pregnant during the COVID-19 pandemic were found to present more psychopathological symptoms such as higher levels of perceived stress than the group who did not experience the COVID-19 pandemic during their pregnancy. The group of women who were pregnant during the pandemic also showed higher levels of perceived stress than the group of women who were pregnant before the pandemic. Consistent with our findings, Mortazavi and Ghardashi [11], pregnant women reported on intense stress the first few weeks of reports of the pandemic in the country because they hoped the pandemic never reached Ghana. These increases in levels of stress among pregnant women during the pandemic may be caused by uncertainty due to lack of knowledge on their health and the baby [43], a high rate of disease transmission, a high mortality rate, and, as a result, dread of contracting the illness that affects both the mother and fetus [8, 44].

In the wake of the pandemic, several restrictions that were introduced such as the lockdown and social distancing protocols targeted at reducing the spread of the disease, however impeded ability to physically interact with others and possibly go on visitations or social functions. Consistent with Atmuri, et al. [39], Zhang and Ma [45], and Kathleen and Sarah [46] our study reported that participants had very good support from family and friends during the period. The safety protocols introduced by the governments had distinct impacts on the citizens socially, as observed in our study. As some enjoyed adequate social support, others had to deal with loneliness and no support because the lockdown restrictions caused inability of their significant others to travel back home. The significance of social support cannot be overlooked considering the positive impact it has on an individual’s psychological functioning. In the case of pregnancy, such support is needed considering the hormonal changes that results in swings in emotions [47, 48]. On the other hand, some participants also reported instances where they received no social support, also presented by findings of Mizrak Sahin and Kabakci [7]. According to them, this lack of support was mainly attributed to the fact that the lockdown and movement restrictions prevented family members from visiting them. As indicated by Atmuri, et al. [39] and Linden, et al. [4], the lack of social support could have contributed to loneliness, anxiety and depressive symptoms.

With regards to the effect on daily routine, it was reported that their routines were unaffected. Most of them had already cut off from visitations and many social functions due to the pregnancy condition. However, Fallon, et al. [35], Mizrak Sahin and Kabakci [7], and Kolker, et al. [43] presented that majority of the participants reported an indirect change in their routines due to the social distancing measures and subsequent lockdown imposition. The contradictions in the findings can be attributed to differences in culture and geographical disparity; in Africa, most families are situated in the same geographical location whereas in most parts of Europe and America, the nuclear families are mostly far away from other relatives.

Many lost their jobs and others could not make enough money to keep their businesses running due to the pandemic. Lockdown imposition also limited trade to only essential services hence financial stress became the order of the day. Women who made a living from provision of non-essential services were left with nothing except the financial support from their significant others. In an instance where their partners were also laid off or had become temporarily unemployed, the financial constraints became unbearable. The financial constraints reported took many forms. These constraints were most likely to affect their patronage of health services and their ability to meet their nutritional requirements. This assertion is consistent with findings of Oluoch-Aridi, et al. [36] where some participants identified economic reasons for not accessing health services. They therefore prioritized essential provisions such as purchasing food over going to the health facility. These forms of financial constraints are most likely predominant among pregnant women who were unemployed or whose partners were also unemployed at the times of the studies.

## 5. Conclusion and recommendations

The current study delved into the psychosocial experiences of pregnant women in the early stages of the COVID-19 pandemic. The findings brought to light various levels of coping during the period. Most women reported high levels of anxiety, often accompanied by feelings of fear. However, the support system during the period is worth appreciating. Economically, pregnant women suffered dearly as they recounted various levels of economic hardships during the pandemic. Based on the findings of our study, the following recommendations have been suggested;

- Healthcare services should be adaptable to meet the changing needs of pregnant women during pandemics. This includes the option for virtual consultations, and enhanced safety measures in healthcare settings.
- There should be counselling services for pregnant women especially during pandemics to provide emotional/psychological support during those periods of uncertainty.

### 5.1 Limitations of the study

- The study is a qualitative study and findings cannot be generalized to the larger population.
- Being a retrospective study, it relies on participants’ recollections of past experiences, which is subject to recall bias.
- The study included only participants who could speak English or Asante Twi. This criterion might exclude non-speakers of these languages, potentially overlooking the experiences of a segment of the population.

### 5.2 Key points for policy, practice and/or research

- A robust support system for pregnant women is necessary in controlling psychopathological symptoms in pregnancy during uncertain times like a pandemic.
- Provision of information through health education is ideal in managing rumor generated fear that arises during a pandemic.
- Hospital policies must take into consideration pregnant women in genuine financial constraints as such groups are more likely to prioritize basic necessities such as nutritional requirements over healthcare patronage.
- Given the heightened perceived stress and anxiety among pregnant women during the pandemic, there should be a policy push for integrating mental health services into prenatal care.

## Supporting information

S1 File Interview guide. (DOCX)

S2 File Interview transcript. (DOCX)
