# Supplementary material for: The psychosocial experiences of pregnant women in the early stages of the COVID-19 pandemic: A retrospective qualitative study
Source: PLoS One. 2024 Feb 28;19(2):e0299219. doi: 10.1371/journal.pone.0299219 (PMC10901345; doi:10.1371/journal.pone.0299219)
Supplement: S1 File — (DOCX) [file pone.0299219.s001.docx]

## **INTERVEIW GUIDE**

1. Please tell me about yourself

- Age of the mother
- Age of infant
- Employment status
- Marital status
- Religion of mother

1. How did you feel when you first heard of the outbreak of COVID-19in Ghana?
2. Did you feel nervous about your pregnancy condition in the wake of the pandemic?

Please give reasons for your answer

1. How would you describe your pregnancy amidst COVID-19 in relation to stress?
2. What were your normal daily activities and how were they affected by the pandemic?
3. How did these changes affect you as a person?
4. How supportive was your family during the COVID-19 period?
5. How was it adhering to the COVID-19 safety protocols in terms of

- Wearing of the nose mask
- Visiting/hosting relatives and friends
- During the lockdown (if applicable)

1. Please share with us, how your work was affected?
2. How were you coping financially?
3. Was your partner working?

If yes, how did you feel about your partner working during the period?

Give reasons for your answer
