# Supplementary material for: The psychosocial experiences of pregnant women in the early stages of the COVID-19 pandemic: A retrospective qualitative study
Source: PLoS One. 2024 Feb 28;19(2):e0299219. doi: 10.1371/journal.pone.0299219 (PMC10901345; doi:10.1371/journal.pone.0299219)
Supplement: S2 File — (DOCX) [file pone.0299219.s002.docx]

**INTERVEIW TRANSCRIPT**

**(**CODE **: Bold= question,** *Italics= answers*)

# RESPONDENT 1

## **DEMOGRAPHICS**

Age of the mother: 28 years

Gravida: 3

Parity: 3

Age of infant: 4 months

Employment status: Unemployed

Marital status: Single

Religion of mother: Christian

## **PSYCHOLOGY IMPACT**

**How did you feel when you first heard of the outbreak of covid-19 in Ghana?**

*I was a bit alarmed, but I wasn't... it was normal, we were all in shock*

**What were your concerns about the Covid-19 outbreak, what caused the alarm?**

*The rate at which people were dying.*

**Did you ever feel that Ghana will not be able to cope with it?**

*NO, I knew it wouldn't survive very long in this country*

**Please what about when you got pregnant during the COVID pandemic?**

*I was okay.*

**You had no concerns, maybe COVID-19 could have some complications your pregnancy?**

*No, I did some background check and I got to find out that it is an airborne disease so if you are able to protect yourself you wouldn't contract it, you'll be safe.*

**How would you describe your pregnancy in terms of stress?**

*It was stressful, wearing of nose masks because during pregnancy breathing isn't comfortable.*

## **SOCIAL IMPACT**

**What were your normal daily activities and how were they affected by the pandemic?**

*I was laid off because of COVID-19, so all my activities were just home. The rate at which i go out to buy stuffs reduced, I buy in bulk unlike before.*

**How did these changes affect you as a person?**

*I was restrained financially*

**How did you cope? Did you have other things to fall on?**

*I realized other potentials and opportunities around me. I started thinking about my own delivery service and yet to start*.

**How supportive was your family during the COVID-19 period?**

*Yes, they helped getting stuff, buying food etc.*

**Did you contract covid?**

*No*

**How was it adhering to the COVID-19 safety protocols in terms of:**

**A. Wearing of the nose mask**

*It was difficult.*

**B. Visiting/hosting relatives and friends**

*It was really affected, unlike before I couldn’t visit my daughter who lives with my mother.*

## **ECONOMIC IMPACT**

**Please share with us, how your work was affected?**

*My work collapsed; I was laid off.*

**How were you coping financially?**

*It was stressful, but later was comfortable.*

# RESPONDENT 2

## **DEMOGRAPHICS**

Age of the mother: 26 years

Gravida: 1

Parity: 1

Age of infant: 5 months

Employment status: Employed

Marital status: Married

Religion of mother: Christian

## **PSYCHOLOGY IMPACT**

**How did you feel when you first heard of the outbreak of COVID-19 in Ghana?**

*I was sad*

**What were your concerns about the COVID-19 outbreak, what caused the alarm?**

*Our health system is very bad*

**Did you ever feel that Ghana will not be able to cope with it?**

*Yes*

**Please what about when you got pregnant during the COVID pandemic?**

*I was happy. I could slow down, plan how to take care of the baby*

**You had no concerns, maybe COVID-19 could have some complications your pregnancy?**

*No, I did some background check and I got to find out that it is an airborne disease so if you are able to protect yourself you wouldn't contract it, you'll be safe.*

**How would you describe your pregnancy in terms of stress?**

*It was not stressing for me, I had it easy by the grace of God*

**What contributed to it not being stressful?**

*We shifted from the normal work schedule and I got more breaks*

## **SOCIAL IMPACT**

**What were your normal daily activities and how were they affected by the pandemic?**

*No effect*

**How did these changes affect you as a person?**

*I realized in life you have to plan a lot, there isn't much time*

**How did you cope? Did you have other things to fall on?**

*I was by myself*

**How supportive was your family during the COVID-19 period?**

*I didn't get support I did it all on my own*

***Did you contract covid?***

*No*

**How was it adhering to the COVID-19 safety protocols in terms of:**

**A. Wearing of the nose mask**

*it was normal*

**B. Visiting/hosting relatives and friends**

*It has no effect; I don't get visits before covid.*

**Did you get pregnant during the lock down?**

*No*

**During the lockdown how were you affected?**

*I was not affected, though one day i had stomach upset and i returned home to take ORS, i was marked absent upon return*

## **ECONOMIC IMPACT**

**Please share with us, how your work was affected?**

*My work was not affected*

**How were you coping financially?**

*My husband was not really affected*

**Was your partner working?**

*He was home but returned to work later.*

**How did you feel about your partner working during the period?**

*It was mixed, was happy because money was coming but concerned about him getting COVID-19*

# RESPONDENT 3

## **DEMOGRAPHICS**

Age of the mother: 28 years

Gravida: 3

Parity: 3

Age of infant: 7 months

Employment status: Employed

Marital Status: Married

Religion of mother: Christian

## **PSYCHOLOGY IMPACT**

**How did you feel when you first heard of the outbreak of covid 19 in Ghana?**

*I thought Ghana could not contain it, even the western world was struggling with deaths*

**Did you feel nervous about your pregnancy condition in the wake of the pandemic?**

*No*

**Please give reasons for your answer**

*Everything was normal*

**How would you describe your pregnancy amidst COVID-19 in relation to stress?**

*It wasn’t stressful but wearing the mask is difficult*

## **SOCIAL IMPACT**

**What were your normal daily activities and how were they affected by the pandemic?**

*I can go for a gathering, meet people, shake hands and the likes of other social activities.*

**How did these changes affect you as a person**?

*I was indoors mostly*

**How supportive was your family during the covid 19 period?**

*They very were supportive*

**Did you contract covid?**

*no*

**How was it adhering to the COVID-19 safety protocols in terms of**

**A. Wearing of the nose mask**

*it was difficult, breathing in pregnancy was already difficult*

**B. Visiting/hosting relatives and friends**

*there was no visiting*

## **ECONOMIC IMPACT**

**Please share with us, how your work was affected?**

*I was home during lockdown*

**How were you coping financially?**

*As a government worker salary was still coming*

**Was your partner working?**

*Yes*

**How did you feel about your partner working during the period?**

*I complained, it was difficult to accept that his work was not running smoothly as before*

# RESPONDENT 4

## **DEMOGRAPHICS**

Age of the mother: 26 years

Gravida: 1

Parity: 1

Age of infant: 2 months

Employment status: Employed

Marital status: Married

Religion of mother: Christian

## **PSYCHOLOGY IMPACT**

**How did you feel when you first heard of the outbreak of covid-19 in Ghana?**

*o, it wasn't easy but, I like I try to protect myself without getting the diseases and at the same time i was pregnant so I had to protect myself and protect my baby.*

**Was it that you were scared or l how were you just feeling?**

*I was not too much scared because of the protection, the hand sanitizer and the nose mask so I was not too much skilled(scared) but the thing is I have to be protected and protect my going out I have to like because of the bolt and Uber and so always pick Uber when coming going somewhere or going to the hospital. I don't like sitting in the trotro because of the spread of this disease.*

**Was there ever a time you felt like Ghana could not handle the covid virus, could not contain it?**

*I don't really know about that, because of we went on national lockdown, right? so I then (nu) it was very serious that’s why we went on national lockdown it was good that we were (like) we went on lockdown to protect us and to protect the whole nation. That helped us, without that then I don't think that we could survive.*

**Did you also ever feel that maybe the covid-19 could cause complications on your pregnancy?**

*Yes. It is a deadly disease and at the same time i am pregnant with my first child so i have to do everything to protect myself to protect the child too.*

**Would you say that covid-19 has put some stress on you?**

*No*

**How would you describe your pregnancy amidst COVID-19 in relation to stress?**

*Not stressful*

## **SOCIAL IMPACT**

**Your normal daily activities before covid-19 was there any change?**

*mmmm NO, like me I’m an indoor person so it like it didn’t change anything just going to the hospital coming back, going to work that’s all, yeah.*

**Was there any change to you as a person related to covid-19 like did it bring you any change, maybe a new perspective of life or anything**?

*I just brought, this is a lockdown baby oo, so this my child changed my life it has given my joy and am okay with it. This is my joy; my child is my joy.*

**How supportive was your family during the covid-19 period?**

*They were very supportive because going out and coming in, washing all your hands, with the nose mask, sanitizer they want to make sure everything is intact before I step out and coming back, they want to be okay before like when I enter in the house, they want me to wash my hands, sanitize my hands before I touch anything.*

**Were they around you?**

*My mom was around me every day and night because i was having a little complication so she was always around me.*

**Did you contract covid?**

*no*

**How was it adhering to the COVID-19 safety protocols in terms of:**

**A. Wearing of the nose mask?**

*it helped me too; it helped me because errr I didn't want to be identified in my early stages of pregnancy so covering of my nose not everyone could see your real face so it really helped me a lot. I needed to protect myself and protect the child for some months before people get to know that i am pregnant. So, with the covering of the face it really helped, when you are walking nobody can identify you quickly. Maybe the person knows you well that on the person can identify you but no everybody could identify that it’s you. With the nose make it really helped me.*

**Why didn't you want people to identify you?**

*It was instruction given to me by my mom, given to me by my pastor so I was trying my best to protect myself and protected the life of my child.*

**B. Visiting/hosting relatives and friends?**

*like me visiting or they visiting me? ooh you talk on phone you have so many video calls and we also talk on phone so it was okay. Just that you are not seeing the person physically. The only place I miss so much was church*

**The lockdown itself what was the impact on you?**

*I’m an indoor person so NO change.*

## **ECONOMIC IMPACT**

**Please share with us, how your work was affected?**

*not applicable*

**You were coping financially, i guess?**

*Yes.*

**Was your partner working?**

*Yes.*

**How did you feel about your partner working during the period?**

*It wasn't easy but I have to let him go.*

# RESPONDENT 5

## **DEMOGRAPHICS**

Age of the mother: 25

Gravida: 2

Parity: 2

Age of infant: 11 months

Employment status: Employed

Marital status: Married

Religion of mother: Christian

## **PSYCHOLOGY IMPACT**

**How did you feel when you first heard of the outbreak of covid-19 in Ghana?**

*We all felt bad because of the death that was happening at the outside and even within Ghana here it wasn't easy. The economy came down and a whole lot of, so we were and even when you go outside you think maybe you’ll also be infected by the virus*.

**Did you ever feel like maybe Ghana may not be able to contain the virus?**

*For me dier, I strongly believe that we will not contain if we follow the rules and the regulations given to us.*

**How did you feel about your pregnancy amidst the pandemic?**

*We were all scared because coming now to the hospital they were even saying that some of the pregnant women have been infected. So, when we come here the way they take care of us wasn’t like the normal time, they had to divide us.*

**How would you describe your pregnancy amidst COVID-19 in relation to stress?**

*I didn’t send my mind there. Am coming to ante natal, am taking my medicine given to me, am doing what am supposed to do. Washing my hands and other things.*

## **SOCIAL IMPACT**

**What were your normal daily activities and how were they affected by the pandemic?**

*No change*

**How did these changes affect you as a person?**

*not applicable*

**How supportive was your family during the covid-19 period?**

*They praying for me, they always advise me to take very good care of myself when am coming to ante natal I have to wear my nose mask, wash my hand use the sanitizer, social distance.*

**Did you contract covid?**

*no*

**How was it adhering to the COVID-19 safety protocols in terms of:**

**A. Wearing of the nose mask**

*When you wear it is not easy, even breathing kraa it becomes, you know pregnancy we are not of ourselves and wearing of this it wasn’t easy but you have to try and wear it for safety.*

**B. Visiting/hosting relatives and friends**

*There was no visiting, we communicated on phone.*

## **ECONOMIC IMPACT**

**Please share with us, how your work was affected?**

*We stayed home for almost 1 year. The kids were home and were not learning, when they resume we are going to suffer. But am back to work.*

**How were you coping financially?**

*Am a government worker so every month my salary comes.*

**Was your partner working?**

*He was at home*

# RESPONDENT 6

## **DEMOGRAPHICS**

Age of the mother- 28 years

Gravida- 1

Parity - 1

Age of infant - 3 months

Employment status - Unemployed

Marital status- Married

Religion of mother- Christian

## **PSYCHOLOGY IMPACT**

**How did you feel when you first heard of the outbreak of covid 19 in Ghana?**

*I didn’t feel anything. I was just sad couldn’t do what I was planning to do especially buying the midwifery form(university).*

**Did you feel nervous about your pregnancy condition in the wake of the pandemic?**

**Please give reasons for your answer**

*I didn’t feel anything. No, I was not nervous it (covid 19) can cause complication to my pregnancy. I had a lot of rets. I wasn’t stressed at all. Just eating, sleeping. I wasn’t working.*

**How would you describe your pregnancy amidst COVID-19 in relation to stress?**

*I wasn’t stressed at all. I would rate my pregnancy 9/10. The 1 is because I had preeclampsia at the very end because my baby was too big.*

## **SOCIAL IMPACT**

**What were your normal daily activities and how were they affected by the pandemic?**

*It involves sleeping, walking and so on. The pandemic didn’t affect them. It rather helped me*

**How did these changes affect you as a person?**

*My pregnancy it affected me in the way that I was supposed to be in school but now I have to wait till he(baby) starts working. I didn’t go the places I wanted to go to due to the pandemic. I was always in bed.*

**To what extent did you receive support from your family?**

*They were very supportive*

**Did you contract covid?**

*No*

**How was it adhering to the COVID-19 safety protocols in terms of:**

1. **Wearing of the nose mask**

*It was stressful. Because you can wear this thing, right now am not feeling comfortable but I have to wear it*

1. **Visiting/hosting relatives and friends**

*It affected everything cause right now I can’t go and see them because am afraid I might contract the disease*

## **ECONOMIC IMPACT**

**How were you coping financially?**

*I worked at marketing company even if I don’t go to work, they still pay me.*

**Was your partner working?**

*No, he is currently schooling.*

# RESPONDENT 7

## **DEMOGRAPHICS**

Age of the mother- 25 years

Gravida - 2

Parity - 2

Age of infant - 10months

Employment status - Employed

Marital status- Single

Religion of mother- Christian

## **PSYCHOLOGY IMPACT**

**How did you feel when you first heard of the outbreak of covid 19 in Ghana?**

*I didn’t think it was real. Because we just heard rumours while we were still in school. When the lockdown happened that is when it dawns on me that it is real. It made a lot of things come to a halt. A lot of things couldn’t happen. Some businesses collapse because of that. Others lost their jobs because of that. So, it was not easy. Especially for those of them in the private sector. I had so many friends who had to be laid off because of the pandemic. They couldn’t pay them so they had to be laid off and it was not easy for them at all. Myself I am not in the private sector.*

**Did you feel nervous about your pregnancy condition in the wake of the pandemic?**

**Please give reasons for your answer**

*When I got pregnant, I was in the house oh. I was just resting. I had a whole year to rest. I came to the house on the 19^th^ of march. I couldn’t go back again. I went back on the 15^th^ of May, 2021. So, for me, during the pandemic I had time to rest during the pregnancy period. Had it not been the pandemic, I would be in schools. I didn’t even think that covid could have some impact on my pregnancy. The only thing is that when I was going for antenatal, that’s when it will come to my mind that EEiih covid is out there out. That’s when it comes to my mind. But God being so good I went through my pregnancy without any blemish.*

**How would you describe your pregnancy amidst COVID-19 in relation to stress?**

*It wasn’t stressful because I was in the house. The only thing stressful was that I would go to antenatal but I will come back and rest. That’s it. I didn’t have anything to do.*

## **SOCIAL IMPACT**

**What were your normal daily activities and how were they affected by the pandemic?**

*I didn’t have anything to do so I was just praying God help me deliver this baby. Then I will relax. Wake up and do the normal things. Watch television and do the normal chores. That all. I was just thinking about my kid oh. Because he is in the village, he doesn’t usually learn.*

**To what extent did you receive support from your family?**

*My family was very supportive of me when I was pregnant.*

**Did you contract covid?**

*No*

**How was it adhering to the COVID-19 safety protocols in terms of?**

1. **Wearing of the nose mask**

*It wasn’t easy. Sometimes you can’t even breathe but you have to force yourself and wear it because you were supposed to wear it. The whole day you will be panting. A pregnant woman with nose mask is not easy. You will be sweating like you have ran for 2000metres!*

1. **Visiting/hosting relatives and friends**

*Not much. They were not even coming because of the lockdown. Our movements were restricted.*

## **ECONOMIC IMPACT**

1. **Please share with us, how your work was affected?**

*Because of the pandemic. I had to stop working*

1. **How were you coping financially?**

*Financially it was not easy but we managed. Since we are Ghanaians, we are all managing, we managed our salary.*

# RESPONDENT 8

## **DEMOGRAPHICS**

Age of the mother- 29

Gravida – 1

Parity - 1

Age of infant - 6 months

Employment status - Employed

Marital status- Married

Religion of mother- Christian

## **PSYCHOLOGY IMPACT**

1. **How did you feel when you first heard of the outbreak of covid 19 in Ghana?**

*It was quite scary since I knew nothing about it and lots of people were dying.*

**Did you ever at some point feel that maybe Ghana couldn’t handle the pandemic?**

*Well I think we hadn’t reached that amount. We were all praying it won’t reach that point but before we knew all of a sudden it was like getting to thousand there about. We were all hoping that it won’t reach Ghana but when it did It was scary when I heard people were dying because of the virus.*

1. **Did you feel nervous about your pregnancy condition in the wake of the pandemic?**

**Please give reasons for your answer**

*Sure, I was, because at that time when I was pregnant, I was still working. although I was abiding by the protocol, washing my hands but yet I was worried because of the patient who comes in and out so that makes me worried that time. Well I never felt covid 19 could have complications on my pregnancy. I heard pregnant women our immune system is low when it comes to this because. (laughs) we were all hoping I won’t get the virus but I was not scared so I knew I won’t get it*

1. **How would you describe your pregnancy amidst COVID-19 in relation to stress?**

*It was quite stressful. Wearing of the mask was a whole lot. I can’t even breathe properly with it. Sometimes I had to just remove the nose mask for a while. That was the only difficult thing for me*

**In terms of your mental health:** *I was quite depressed because I said this era getting pregnant is a whole lot. I could have wait for the next year when covid is gone but….*

## **SOCIAL IMPACT**

1. **What were your normal daily activities and how were they affected by the pandemic?**

*I used to sell things in the house but when covid came I couldn’t go for my goods and all that. The covid prevented me from doing other business that I do usual*

1. **How did these changes affect you as a person?**

*It affected me financially.*

1. **To what extent did you receive support from your family?**

*My family was around. If you buy food stuff in the house, so at least if you don’t have much money on you, you can just take some. We were just cooking. I got support from friends especially at church. At church I quite remember they provided rice and oil. They also did well.*

1. **Did you contract covid?**

*No*

1. **How was it adhering to the COVID-19 safety protocols in terms of :**
2. **Wearing of the nose mask**

*It was stressful*

1. **Visiting/hosting relatives and friends**

*At least they made phone calls and all that but the visit was normal. Its like you have a family but you can’t reach out.*

**C. During the lockdown (if applicable).**

*It was like being encamped in one place*

## **ECONOMIC IMPACT**

**Please share with us, how your work was affected?**

*I used to go every day but when the virus came, I used to go 2 days then we had 3 days off. It was good because at least you can get some day off but the bad part is going to work knowing the virus is there, sitting in a car, I quite remember when I was sitting in the car and someone will just cough on your back, YIE. And sometimes people will bring their children to the hospital and the temperature is like 28 degree Celsius.*

**You were coping financially, I guess, since you were working**

*Yeah, I was coping*

**Was your partner working?**

*Yes, he was working. I wished we all stayed in the house and we survive on the little we have but here is the case we can’t do that. We were just praying to God nothing happens to us.*

# RESPONDENT 9

## **DEMOGRAPHICS**

Age of the mother- 30

Gravida – 3

Parity - 3

Age of infant - 10 months

Employment status - Employed

Marital status- Married

Religion of mother- Christian

## **PSYCHOLOGY IMPACT**

1. **How did you feel when you first heard of the outbreak of covid 19 in Ghana?**

*It was scary with the numbers raising every day. But then the lockdown occurred in Accra so I had to come to Kumasi to be with my family. There was panic. You don’t know the next person standing with you. Everyone was a suspect*

1. **Did you feel nervous about your pregnancy condition in the wake of the pandemic?**

**Please give reasons for your answer**

*I found out I was pregnant in September so it had subsided a little. There was calm. The only time I felt disappointed was when the vaccine was available and I couldn’t take it because I was pregnant. Aside that I was ok. There were no complications during my pregnancy.*

1. **How would you describe your pregnancy amidst COVID-19 in relation to stress?**

*It was stressful but it won’t be related to covid. I was moving around, working*

## **SOCIAL IMPACT**

1. **To what extent did you receive support from your family?**

*I have a big family. It was me, my husband, our three kids, our niece, and my husband friend. I wasn’t lonely at all. They were supportive.*

1. **Did you contract covid?**

*No*

1. **How was it adhering to the COVID-19 safety protocols in terms of :**
2. **Wearing of the nose mask**

*It wasn’t easy. But I had to wear it to protect myself and my baby*

## **ECONOMIC IMPACT**

1. **Please share with us, how your work was affected?**

*I work in Accra. The toughest aspect of covid was that I am on a project and I had come to Kumasi for data and there was lockdown plus covid. So, I had to wait for about a month to get my participant me coming over or meeting you at your place. It was a little inconvenience for me and then. I was also pregnant. I had to go back to Accra. In January-March. There were a second wave that time so it was hard.*

1. **Was your partner working?**

*No*

# RESPONDENT 10

## **DEMOGRAPHICS**

Age of the mother- 30 years

Gravida – 2

Parity - 2

Age of infant (last baby)- 12 months

Employment status - Employed

Marital status- Married

Religion of mother- Christian

## **PSYCHOLOGY IMPACT**

1. **How did you feel when you first heard of the outbreak of covid 19 in Ghana?**

*I was very afraid cause I thought I will get some*

1. **Did you feel nervous about your pregnancy condition in the wake of the pandemic?**

**Please give reasons for your answer**

*I thought in pregnancy I won’t get covid. When you are pregnant, they are saying you are heating already I heard a story that covid 19 don’t like heat because of Ghana its hots. So, I drink hot water and take a lot of pepper. I like pepper. I heard covid 19 don’t like pepper.*

1. **How would you describe your pregnancy amidst COVID-19 in relation to stress?**

*It was not stressful. I was resting at home*

## **SOCIAL IMPACT**

1. **What were your normal daily activities and how were they affected by the pandemic?**

*For my daily activities I go market and buy stuffs but I wore the mask. My daily activities weren’t that affected*

1. **To what extent did you receive support from your family?**

*They were very supportive*

1. **Did you contract covid?**

*No*

1. **How was it adhering to the COVID-19 safety protocols in terms of :**
2. **Wearing of the nose mask**

*I had difficulty breathing with the nose mask*

1. **Visiting/hosting relatives and friends**

*I was alone at home with my husband.*

## **ECONOMIC IMPACT**

1. How were you coping financially?

*My partner was working and I was comfortable with it*

# RESPONDENT 11

## **DEMOGRAPHICS**

Mother’s age- 29 years

Gravida – 2

Parity - 2

Age of infant - 9 months

Employment status - Employed

Marital status- Married

Religion of mother- Christianity

## **PSYCHOLOGICAL IMPACT**

**How did you feel when you first heard the outbreak of COVID-19 IN Ghana?**

*I was not really scared because I was always in the house because of my pregnancy. I had a strong hope in God that none of my relatives would be infected and bring it to the house. I’ve always believed God is the one in control. No amount of effort made by a human being in order not to contract the disease would become a success if God says that person will contract it. Afterall, my family and I are not better than the people who contracted the disease. Due to these reasons, I had no fears at all. Even at a point within the early stages of the pandemic, I was hospitalized for two months because of pregnancy complications so I was thinking about my life not Covid-19 infection.*

## **SOCIAL IMPACT**

**What were your normal daily activities and how were they affected by the pandemic?**

*The doctor ordered me not to do anything in the house because of my pregnancy complications so I was always in bed. Even when I wanted to bath, they had to carry my water into the bathroom. In view of this, I will not say the pandemic affected my normal daily activities.*

**To what extent did you get support from your family?**

*My family supported me in basically everything. Like I said, they even carried my water to the bathroom when I wanted to bath*.

**Did you contract covid?**

*No*

**How was it adhering to the COVID-19 safety protocols in terms of;**

**A. Wearing of nose mask?**

*I wasn’t wearing the nose mask at all because I wasn’t going out and I also got hospitalized for a long time so there was no need for the nose mask. My condition was just not the best.*

**B. Visiting/hosting relatives?**

*I had most of my relatives around so there was no need going for a visit. My condition couldn’t have permitted me though.*

## ECONOMIC IMPACT

**Please share with us how your work was affected.**

*I closed down my shop because of the pregnancy complications and not really because of the pandemic even though I know I would have still closed the shop because of the pandemic.*

**So how were you coping financially?**

*I was solely depending on my husband. He was the one doing everything in terms of finances.*

**Was your partner working?**

*Yes, he was working*

**How did you feel about your partner working during that period?**

*I was very certain he was not going to be infected because he’s a very cautious person. In fact, he was more scared than I was so he was religiously adhering to the safety protocols.*

# RESPONDENT 12

## **DEMOGRAPHICS**

Mother’s age- 30 years

Gravida – 1

Parity - 1

Age of infant - 6 months

Employment status - Employed

Marital status- Married

Religion of mother- Christianity

## **PSYCHOLOGICAL IMPACT**

**How did you feel when you first heard the outbreak of COVID-19 in Ghana?**

*I was afraid because from the news, we heard it was killing people outside Ghana.*

**Did you feel nervous about your pregnancy condition in the wake of the pandemic?**

**Please give reasons for your answer**

*I didn’t really think it could affect my pregnancy because of the safety measures the government put in place. I was also a regular ANC attendant and because of that I didn’t feel nervous at all.*

**How would you describe your pregnancy amidst COVID-19 in relation to stress?**

*I would say it was very stressful because of the restrictions or safety protocols. Not going to places, washing hands frequently and especially wearing the nose mask.*

## **SOCIAL IMPACT**

**What were your normal daily activities and how were they affected by the pandemic?**

*Because we teachers and our students were made to stay home, I used to visit the workers at my cosmetic shop a lot. That was what I was doing almost every day but I think the pandemic negatively affected my shop because we were not really getting clients.*

**How did these changes affect you as a person?**

*Going to the cosmetic shop helped me to release stress considering the fact that I wasn’t going to work anymore.*

**How supportive was your family during the covid 19 period?**

*The only family member that was close was my husband and he used to help me with the house chores.*

**Please did you contract COVID-19.**

*Not at all*

**How was it adhering to the COVID-19 safety protocols in terms of;**

**A. Wearing of the nose mask**

*Initially, wearing the nose mask in my pregnancy was so unbearable because I could not breath but as time went on, I became used to it*

**B. Visiting/hosting relatives and friends**

*I used to talk to friends and relatives on phone so I wasn’t bothered because I was not seeing them.*

## **ECONOMIC IMPACT**

**Please share with us, how your work was affected?**

*The government closed down all schools as part of the measures put in place to curb the pandemic and because of that I had to stay home even as a teacher.*

**How were you coping financially?**

*Oh! Like I said, I’m a government school teacher so the government was still paying us at the end of every month even as we were home. My husband too was fulfilling his financial commitment to me.*

**Was your partner working?**

*Yes, he was working but had to stay home during the lock down. After the lock down, he resumed work.*

**If yes, how did you feel about your partner working during the period?**

**Give reasons for your answer**

*I was scared for him but because he was adhering to all the safety protocols, my heart became at ease.*

# RESPONDENT 13

## **DEMOGRAPHICS**

Age of the mother- 27 years

Gravida – 2

Parity - 2

Age of infant- 8 months

Employment status - Unemployed

Marital status- Married

Religion of mother- Christianity.

## **PSYCHOLOGY IMPACT**

**How did you feel when you first heard of the outbreak of covid 19 in Ghana?**

*“I was really scared because the media was reporting that most Italians were dying of the pandemic. The thought of it always gave me headaches seriously.”*

**Did you feel nervous about your pregnancy condition in the wake of the pandemic?**

**Please give reasons for your answer**

*“Oh! Not really because after the lock down most people stopped panicking including me. This was because safety measures had been laid down and there was enough education about the pandemic and how one could prevent oneself from being infected.”*

**How would you describe your pregnancy amidst COVID-19 in relation to stress?**

“*This pregnancy was really stressful because one had to be always covering one’s nose and mouth, washing hands almost every time and distancing oneself from people all the time. But generally speaking, I’ll say all these protocols also made me very hygienic and protected me from other sicknesses, not only COVID-19.”*

## **SOCIAL IMPACT**

**What were your normal daily activities and how were they affected by the pandemic?**

*“Because I lost my job as a result of the pandemic, I was always home doing normal house hold chores like cleaning, cooking, washing and taking care of my first child. I’ll say the pandemic positively affected my normal daily activities.”*

**How did these changes affect you as a person?**

“*The changes made me a better mom and wife because it offered me more time for my son and husband.”*

**How supportive was your family during the covid 19 period?**

“*My husband used to help me in doing my chores especially washing. And he’s the only relative and support I have.”*

**Please did you contract COVID-19.**

*“Not at all. But who would tell you if they contacted the disease? Nobody will do that because they will be stigmatized. I didn’t contract it though.”*

**How was it adhering to the COVID-19 safety protocols in terms of;**

**A. Wearing of the nose mask**

*“Seriously it wasn’t easy for me and because of that I wasn’t even going to church. Even when I went to the ANC, I’ll shit the nose mask on my nose and when I’m entering the consultant room, I’ll properly wear it other than that I’ll not be attended to by the doctor. When I’m going to sit in a commercial car, I wear it despite the stress and the difficulty breathing in order to protect myself.”*

## **ECONOMIC IMPACT**

**Please share with us, how your work was affected?**

“*Because our work got spoilt, I had to come and stay home and till now, I’ve not had another job. I’ll say the pandemic has rendered me unemployed.*

**How were you coping financially?**

*“Before I lost my job, I had already saved enough money that could sustain me so I was depending heavily on my savings and my husband too supported financially.”*

**Was your partner working?**

“Yes, he was working.”

**If yes, how did you feel about your partner working during the period?**

**Give reasons for your answer**

*“I was really scared for him so I constantly reminded him on a phone call to strictly adhere to the safety protocols and whenever he came from work, we had to make sure he undressed at the entrance, then I would send him soap and water to wash his hands before coming inside the room.”*

# RESPONDENT 14

## **DEMOGRAPHICS**

Age of the mother- 29 years

Gravida – 4

Parity - 3

Age of infant-15 months

Employment status - Employed

Marital status- Married

Religion of mother- Christianity

## **PSYCHOLOGY IMPACT**

**How did you feel when you first heard of the outbreak of covid 19 in Ghana?**

*“I really got scared because my husband who was abroad used to tell me how people were dying at their end.”*

**Did you feel nervous about your pregnancy condition in the wake of the pandemic?**

**Please give reasons for your answer**

*“I was very nervous because I knew pregnancy is a high-risk condition and anything could happen to it if I got infected with COVID-19.”*

**How would you describe your pregnancy amidst COVID-19 in relation to stress?**

*“I’d say pregnancy amidst COVID-19 was very stressful because, my husband could not come down to Ghana which put emotional stress on me. Wearing of the nose masks too was a big deal for me”*

## **SOCIAL IMPACT**

**What were your normal daily activities and how were they affected by the pandemic?**

*“I used to do home chores like cleaning, washing and taking care of my 3 kids and nothing changed about that. Only that, it offered me more time to be with the kids.”*

**How did these changes affect you as a person?**

*“I’d just say it made me a better mother to my kids that’s all.”*

**How supportive was your family during the covid 19 period?**

*“In fact, I had support from no family member because none of them wanted to travel to Kumasi sake of fear of contracting the disease. My husband too couldn’t come because airports were closed. I was very lonely and even bled at a point but no one was there to help. Because the midwives had given us their contacts, I had to call one of them to teach me what to do before reporting to the hospital.”*

**Please did you contract COVID-19.**

*“Oh! No, I didn’t contract COVID”*

**How was it adhering to the COVID-19 safety protocols in terms of;**

**A. Wearing of the nose mask**

“*Like I said earlier, wearing the nose mask was very unbearable for me because I was struggling to breath anytime I wore it.”*

**B. Visiting/hosting relatives and friends**

*“I was neither visiting or hosting friends or family but we used to talk on phone.*

**C. During the lockdown (if applicable)**

*“It wasn’t easy because my husband who had scheduled to come around that period couldn’t make it. I was very lonely and emotionally tortured.”*

## **ECONOMIC IMPACT**

**Please share with us, how your work was affected?**

*“Sake of the pandemic I had to close down my shop for the fear of contracting it from a client. Before I even closed down the shop, client number had reduced drastically because people were not going out, hence no need for make-overs.”*

**How were you coping financially?**

*“I was relying on my husband and my personal savings.”*

**Was your partner working?**

*“Yes, he was working before and after their lockdown”.*

**If yes, how did you feel about your partner working during the period?**

**Give reasons for your answer**

*“I was scared because he could contract the disease. Especially where he was, Italy.”*

# **INTERVIEW 15**

## **DEMOGRAPHICS**

Age of the mother- 29 years

Gravida – 2

Parity - 2

Age of infant- 11 months

Employment status - Unemployed

Marital status- Married

Religion of mother- Christianity

## **PSYCHOLOGY IMPACT**

**How did you feel when you first heard of the outbreak of covid 19 in Ghana?**

*“I was really nervous because of the signs and symptoms it presented with, and how people were dying in other countries.”*

**Did you feel nervous about your pregnancy condition in the wake of the pandemic?**

**Please give reasons for your answer**

*“Yes, because pregnancy itself shortens one’s breath so I was nervous that I could not breath at all if I was infected with the disease, which would have led to my instant death.”*

**How would you describe your pregnancy amidst COVID-19 in relation to stress?**

*“Pregnancy within COVID-19 period was so stressful because of the wearing of nose masks and having to stay home. I’m not used to staying home for a long time because I was working, so when we were made to stay home for that long, I was really stressed.”*

## **SOCIAL IMPACT**

**What were your normal daily activities and how were they affected by the pandemic?**

“*My normal daily activities were doing my house chores and taking care of my son and nothing changed about that. It even gave me more time for my son and husband.”*

**How did these changes affect you as a person?**

*These changes made me a good wife to my husband and a good mother to my son.”*

**How supportive was your family during the covid 19 period?**

*“The only family I have is my husband and he supported me in every step of the way. From helping me to bath the boy to taking me for antenatal check-ups anytime my date was due. As you can even see, he’s here with me.”*

**Please did you contract COVID-19.**

*“Not at all.”*

**How was it adhering to the COVID-19 safety protocols in terms of;**

**A. Wearing of the nose mask**

*“Even though it was difficult wearing it, I still wore it because I wanted to protect myself and my family.”*

**B. Visiting/hosting relatives and friends**

*“I wasn’t visiting anyone neither was someone visiting me but I used to talk to my friends via video calls a lot.”*

## **ECONOMIC IMPACT**

**Please share with us, how your work was affected?**

*I was sales personnel but because of COVID-19, I am not working anymore.”*

**How were you coping financially?**

*My husband was supporting me financially.”*

**Was your partner working?**

“*No, he wasn’t. He is a teacher and had to stay home for about 10 months too.”*
